# The Pattern of Microorganisms and Drug Susceptibility in Pediatric Oncologic Patients with Febrile Neutropenia

**DOI:** 10.1155/2021/6692827

**Published:** 2021-03-29

**Authors:** Thanyathorn Jungrungrueng, Suvaporn Anugulruengkitt, Supanun Lauhasurayotin, Kanhatai Chiengthong, Hansamon Poparn, Darintr Sosothikul, Piti Techavichit

**Affiliations:** ^1^Department of Pediatrics, Faculty of Medicine, Chulalongkorn University, Bangkok, Thailand; ^2^Division of Pediatric Infectious Diseases, Department of Pediatrics, Faculty of Medicine, Chulalongkorn University, Bangkok, Thailand; ^3^STAR Clinical Research for Holistic Management in Pediatric Hematology and Oncology, Department of Pediatrics, Faculty of Medicine, Chulalongkorn University, Bangkok, Thailand

## Abstract

**Objective:**

The study aimed to describe the pattern of causative microorganisms, drug susceptibility, risk factors of antibiotic-resistant bacterial infection, and clinical impact of these organisms on pediatric oncology patients with febrile neutropenia.

**Methods:**

A retrospective descriptive study of oncologic patients aged less than 15 years who were diagnosed with febrile neutropenia in King Chulalongkorn Memorial Hospital was conducted between January 2013 to December 2017. Characteristics and clinical outcomes of febrile neutropenia episodes, causative pathogens, and their antibiotic susceptibilities were recorded.

**Result:**

This study included 267 patients with 563 febrile neutropenia episodes. The median (range) age was 5.1 years (1 month–15 years). The most common underlying disease was acute lymphoblastic leukemia (42.7%). Of 563 febrile episodes, there were 192 (34.1%) with microbiologically documented infection. Among these 192 episodes of microbiologically documented infection, there were 214 causative pathogens: 154 bacteria (72%), 32 viruses (15%), 27 fungus (12.6%), and 1 *Mycobacterium tuberculosis* (0.4%). Gram-negative bacteria (48.6%) accounted for most of the causative pathogens. Twenty-three percent of them were multidrug resistant, and 18% were carbapenem resistant. Among Gram-positive bacterial infection which accounted for 23.4% of all specimens, the proportion of MRSA was 20%. The 2-week mortality rate was 3.7%. Drug-resistant Gram-negative bacterial infection caused significant adverse events and mortality compared to nonresistant bacterial infection (*p* < 0.05).

**Conclusion:**

There is high rate of drug-resistant organism infection in pediatric oncology patients in a tertiary-care center in Thailand. Infection with drug-resistant Gram-negative bacterial infection was associated with significant morbidity and mortality. Continuous surveillance for the pattern of drug-resistant infections is crucial.

## 1. Introduction

Febrile neutropenia is a common treatment-related complication in oncologic pediatric patients with substantial morbidities and mortalities. Recent studies found that the mortality rate of febrile neutropenic pediatric patients was approximately 0.5–6 percent [1–3]. A previous study in King Chulalongkorn Memorial Hospital, Thailand, from 2007 to 2009 found that the incidence of febrile neutropenia in pediatric patients per 100 admissions was 21.2 and the mortality rate was 16.6 percent [4].

Antibiotics remain the mainstay treatment of febrile neutropenia which influences the clinical outcome of infection. The choice of empirical antibiotic treatment should be based on the patient's infectious risk, potential site of infection, predominant causative pathogens, and sensitivity of those organisms to antibiotics which varies between each center [5]. Recent studies have found that 25–50% of febrile neutropenic episodes were microbiologically documented infections [2, 3, 5, 6]. The previous studies during 2000–2010 showed that the most common isolated pathogens in febrile neutropenic patients were bacteria [2–6]. There has been an increasing proportion of Gram-positive bacterial infection due to central venous catheter insertion in these patients [6–8]. Previous studies in developed countries found that there has been a shift in the causative microorganisms to Gram-positive bacteria [2, 6, 7, 9, 10] but Gram-negative bacteria continue to be a predominant causative pathogen among developing countries [3–5, 11] The previous study during 2007–2009 in Thailand reported that the most common pathogens isolated from cultures in pediatric oncologic patients were Gram-negative bacteria (80%) [4].

There is an increasing number of antibiotic-resistant bacterial infections due to inappropriate usage of antibiotics [11, 12]. The incidence of antibiotic-resistant bacterial infection such as multidrug-resistant (MDR) Gram-negative bacteria and methicillin-resistant *Staphylococcus aureus* (MRSA) is different depending on the site and region of the world which influence the selection of antibiotic therapy in febrile neutropenia. The incidence of antibiotic-resistant bacterial infection has dynamically changed over time, and this affects the choices of antibiotic treatments and clinical outcome. The data regarding the incidence, drug-resistant pattern, associated risk factors, and clinical impact of resistant bacterial infection are limited in children with febrile neutropenia. This study was conducted to elucidate the causative microorganisms (bacteria, virus, and fungus) in pediatric oncologic patients with febrile neutropenia and the risk factors of antibiotic-resistant bacterial infection and clinical impact of these organisms.

## 2. Materials and Methods

### 2.1. Study Population and Design

This study was a retrospective descriptive study approved by the Faculty of Medicine, Chulalongkorn University Institutional Review Board (IRB No. 261/61). All children aged less than 15 years with an underlying malignancy who were hospitalized due to febrile neutropenia in King Chulalongkorn Memorial Hospital between 1 January 2013 and 31 December 2017 were reviewed. Patients were identified by using the hospital medical database ICD-10 code D630, D70, and D696, which referred to anemia in neoplastic disease, agranulocytosis, and thrombocytopenia. Patient medical records were reviewed for the following: demographic data, medical history including underlying diseases, remission stage of malignancy, treatment preceding infection, granulocyte-colony-stimulating factor use, steroid use within 14 days prior to admission, clinical course of febrile neutropenic episodes (duration of fever prior to admission, interval since the last chemotherapy, clinical assessment of infectious sources, vital signs, clinical complications, choices of treatment, duration of hospitalization, and treatment outcome), laboratory investigation results including absolute neutrophil count, duration of neutropenia, and microbiological results with blood culture and cultures from other sites, microorganisms identification, and their drug susceptibility pattern.

### 2.2. Clinical Definition

Febrile neutropenia was defined as fever which has a single oral temperature measurement of ≥38.3°C or a temperature of ≥38.0°C sustained over 1 hour, and neutropenia was identified when the absolute neutrophil count (ANC) was <500 cells/mm^3^ or an ANC that is expected to decrease to 500 cells/mm^3^ during the next 48 hours [13].

Febrile neutropenia episodes were classified as microbiologically documented infection, clinically documented infection, and fever of unknown origin [2, 6]. The diagnosis of microbiologically documented infection was isolation of a pathogen from body fluids such as blood, urine, and sputum in the clinical setting of suspected infection. Clinically documented infection was defined as evidence of focal inflammation on physical examination but without microbiological confirmation. Fever of unknown origin was defined as fever without any focus or etiology identified by clinical history, physical examination, and radiological or microbiologic testing. [2, 6].

### 2.3. Microbiological Definition

MDR Gram-negative microorganisms were defined as Gram-negative bacteria with acquired nonsusceptibility to at least one agent in three or more antimicrobial categories [14]. Carbapenem-resistant Gram-negative microorganisms were defined as Gram-negative bacteria that are resistant to one of the carbapenem class of antibiotics [14]. Methicillin-resistant *Staphylococcus aureus* (MRSA), methicillin-resistant coagulase negative *Staphylococci* (MRCoNS), and vancomycin-resistant *Enterococci* (VRE) were defined as antibiotic-resistant Gram-positive bacteria.

### 2.4. Outcome Evaluation

Severe complications included hypoxia, shock, renal dysfunction, hepatic dysfunction, intensive care unit (ICU) admission, receiving mechanical ventilator care, and death. Hypoxia was defined as an oxygen saturation below 90 percent identified by using a pulse oximeter. Shock was defined as a systolic blood pressure below the 5^th^ percentile for an age-matched normal range and received fluid resuscitation or inotropic agents to raise blood pressure. Renal dysfunction was defined as a serum creatinine >0.5 mg/dL, ≥2 times of the upper limit for age, or 2-fold increased from the baseline creatinine value. Hepatic dysfunction was defined as a serum total bilirubin ≥4 mg/dL or alanine transaminase level increased to more than two times of the upper normal limit for age [15].

### 2.5. Statistical Analysis

Categorical variables were expressed as numbers and percentages. Continuous variables were expressed as medians and ranges. Clinical factors and the frequency of complications were compared between antibiotic-resistant and nonresistant bacterial infection. Categorical variables were compared by using Fisher's exact test, and continuous variables were compared by using a Mann–Whitney test. Statistical analysis was performed using SPSS version 21. A *p* value of <0.05 was considered significant.

## 3. Results

### 3.1. Baseline Patient's Characteristics

From January 2013 to December 2017, there were a total of 1,244 admissions with diagnosis of anemia in neoplastic disease, agranulocytosis, and thrombocytopenia from ICD10 code D630, D70, and D696. We excluded 796 admissions due to repeat patients and incompatible history or investigations with febrile neutropenia. Therefore, during the study period, 267 patients with 448 admissions and 563 episodes of febrile neutropenia were included in this study. The median age of the enrolled children was 5.1 years (range: 1 month–15 years), and 151 patients (56.6%) were male. The most common underlying disease was hematologic malignancy (191 patients, 71.5%). Demographic features of the patients are shown in Table 1. The median white blood cell count at the initial assessment was 670 cells/mm^3^ (range: 10–235, 150 cells/mm^3^), and the median absolute neutrophil count was 70 cells/mm^3^ (range: 0–9, 522 cells/mm^3^).

### 3.2. Characteristics of Febrile Neutropenia Episodes

The median interval from the last chemotherapy was 10 days (range: 0–148 days). The median duration of neutropenia was 8 days (range: 1–49 days). The median duration of fever was 3 days (range: 1–38 days). Characteristics of febrile neutropenia episodes are shown in Table 2. From the total number of 563 febrile neutropenia episodes, 279 episodes (49.6%) were categorized as fever of unknown origin, 192 episodes (34.1%) were classified as microbiologically documented infection from an identified pathogen by a laboratory test, and 92 episodes (16.3%) were classified as an infectious etiology from clinical findings without microbiological confirmation (clinically documented infection). Oropharyngeal infection is the most common source of infection in clinically documented infection (26/92; 28%), followed by skin and soft-tissue infection (17/92; 18.5%).  Table 3 summarizes the etiologies and outcomes of febrile neutropenic episodes. The mortality rate within 2 weeks of febrile episodes was 3.7%.

Among 192 microbiologically documented febrile neutropenia episodes, there were 214 positive specimens (16 episodes with 2 positive cultures and 3 episodes with 3 positive cultures). The causative microorganisms are shown in Table 4. The most common identified pathogen was bacteria (154 episodes; 72%) followed by viral pathogens (32 episodes, 15%), fungus (27 episodes, 12.6%), and *Mycobacterium tuberculosis* (1 episode, 0.4%). Gram-negative bacteria were the most common pathogen (48.6%), followed by Gram-positive bacteria (23.4%). Among positive bacterial cultures, the most common bacteria which were isolated were *Escherichia coli* (38/154; 24.7%), *Klebsiella pneumoniae* (19/154; 12.3%), and *Acinetobacter baumannii* (14/154; 9.1%). From positive fungal cultures, the most common isolated pathogens were *Aspergillus* spp. (12/27; 44%) and *Candida* spp. (12/27; 44%).

A majority of positive cultures were from blood culture (92; 43%). The most common isolated pathogens from blood culture were *Escherichia coli* (18/92; 19.6%), coagulase-negative *Staphylococci* (15/92; 16.3%), and *Klebsiella pneumoniae* (14/92; 15.2%). There were polymicrobial infections from 19 febrile neutropenia episodes (3.4%). Febrile neutropenia episodes with polymicrobial infection are shown in Table 5.

**Figure 1:**
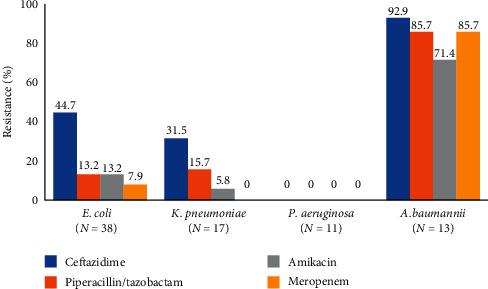
Percentage of antibiotic resistance of Gram-negative bacteria.

**Figure 2:**
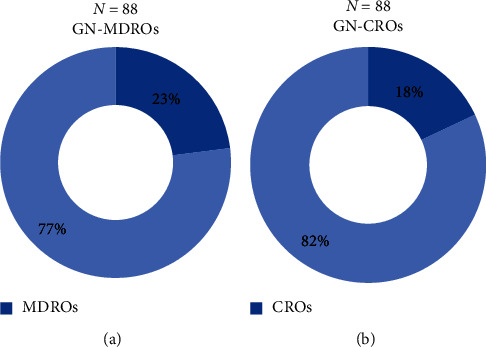
Proportion of Gram-negative multidrug-resistant microorganisms. *∗*Available data of 88/104 cultures. ∗∗GN-MDROs, multidrug-resistant Gram-negative microorganisms; GN-CROs, carbapenem-resistant Gram-negative microorganisms.

**Figure 3:**
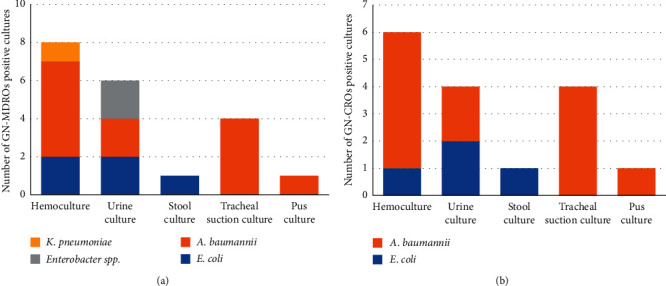
(a) Gram-negative multidrug-resistant microorganisms' positive cultures according to infection sites (*N* = 20). ∗∗GN-MDROs, multidrug-resistant Gram-negative microorganisms. (b) Gram-negative carbapenem-resistant microorganisms' positive cultures according to infection sites (*N* = 16). ∗∗GN-CROs, carbapenem-resistant Gram-negative microorganisms.

We further analyzed the data of antibiotic-resistant bacterial infections. From all Gram-negative bacterial infections (104 cultures), antibiotic susceptibility data were available in 88 cultures. The proportion of MDR Gram-negative microorganisms was 23% (20/88), and the proportion of Carbapenem-resistant Gram-negative microorganisms was 18% (16/88). In consideration of each pathogen, 44.7% of *E. coli* and 31.5% of *K. pneumoniae* were resistant to ceftazidime. No resistance to ceftazidime, piperacillin/tazobactam, amikacin, and meropenem was found among *P. aeruginosa* isolates. Ninety-three percent of *A. baumannii* were resistant to ceftazidime, 70% of them were resistant to amikacin, and 85% of them were resistant to piperacillin/tazobactam and meropenem. The percentage of resistance to antibiotics of each microorganism is shown in Figure 1, and the proportion of drug-resistant Gram-negative bacterial infection is shown in Figure 2. In addition, infection sites of drug-resistant Gram-negative bacterial infection is shown are Figures 3(a) and 3(b). Fifty episodes (23.4%) of the identified pathogens were Gram-positive bacterial infection, of which antibiotic susceptibility data was available in 29 cultures. The proportion of MRSA was 20% (2/10), MRCoNS was 70% (7/10), and VRE was 22.2% (2/9).

### 3.3. Risk Factors and Clinical Impact of Antibiotic-Resistant Gram-Negative Bacterial Infection

Among patients with antibiotic-resistant bacterial infection, the proportion of receiving induction course of chemotherapy was significantly higher than in non-antibiotic-resistant group: 55% vs. 22.6% (*p*=0.006). Patients with antibiotic-resistant bacterial infection had a significantly longer duration of fever after empirical antibiotic therapy when compared with the non-antibiotic-resistant group (15 vs. 7 days, *p*=0.001). In terms of clinical outcomes, renal dysfunction occurred more significantly in antibiotic-resistant bacterial infection when compared with non-antibiotic-resistant bacterial infection, 20% vs. 3.6%, respectively (*p*=0.024). In addition, mortality rates were reported more frequently in antibiotic-resistant bacterial infection: 20% vs. 3.6% (*p*=0.024) (Tables 6 and 7).

## 4. Discussion

Febrile neutropenia is a common complication in oncologic patients after receiving chemotherapy. This study reported that only one-third of febrile episodes were microbiologically documented infection. The most common causative pathogens were bacteria in which Gram-negative bacteria accounted for half of the patients. Among Gram-negative bacterial infection, 23% were multidrug resistant and 18% were carbapenem resistant. Regarding clinical outcomes, admission to the ICU, renal dysfunction, and mortality occurred more significantly in patients with an antibiotic-resistant bacterial infection.

In our study, 34% of febrile neutropenia episodes were microbiologically documented infection, which is consistent with previous studies where pathogens were identified in 22–56% of febrile neutropenic episodes [2, 3, 5, 6]. In microbiologically documented febrile neutropenia episodes, the most common identified pathogen was bacteria (74%). Most were Gram-negative bacteria (48.6%), followed by Gram-positive bacteria (23.4%) and virus (15%), which is similar to previous studies in Thailand and some Asian countries [3–5, 11]. However, the studies in western countries showed an increasing proportion of Gram-positive bacterial infection of up to 60–70% of all microorganisms identified [2, 6, 7, 9, 10] which may be explained by more intense chemotherapy regimens resulting in increased risk of mucositis, frequent use of central venous catheters, and use of antibiotics prophylaxis during neutropenia [2, 3, 6, 16].

The most common isolated bacteria in our study were *E. coli* (24.2%), *K. pneumoniae* (12.3%), and *A. baumannii* (9.1%). Our results of the causative pathogens are different from other previous studies in the type and frequency of pathogens [2–6]. Therefore, the empirical antibiotic therapy in febrile neutropenia should be the agents that have activity against both Gram-negative and Gram-positive bacteria, and it should be adjusted based on the predominant causative pathogen in each treatment center.

The majority of positive cultures were from blood culture, which was similar to the previous studies [2, 5, 6].

A previous study found that 12% of positive hemoculture were polymicrobial infection [2]. However, 3.4% of all febrile neutropenic episodes in our study were polymicrobial infection with coinfection involving bacteria, virus, and fungus isolated from clinical specimens. Polymicrobial infection may affect the clinical outcome of febrile neutropenic episodes.

The proportion of antibiotic-resistant bacteria tends to increase among both Gram-negative and Gram-positive bacterial infection. We found that approximately 40% of *E. coli* were resistant to third-generation cephalosporin, an increase from our previous studies (34%) and data from the study of the National Antimicrobial Resistant Surveillance, Thailand (NARST), which reported a resistance of 30% [4, 17], while it was found that 90% of *E. coli* were susceptible to piperacillin/tazobactam, amikacin, and carbapenem, which was similar to previous studies in Thailand [4, 17]. The percentage of resistance of *K. pneumoniae* to third-generation cephalosporin was similar to previous studies (30–40%) [4, 17]. No drug-resistant *P. aeruginosa* were reported in our study. On the contrary, over 15% of *P. aeruginosa* in the study of the NARST were drug resistant. The percentage of resistance of *A. baumannii* to third-generation cephalosporin, piperacillin/tazobactam, amikacin, and carbapenem from this study was 85–90%, which was higher than in previous studies in Thailand (70–80%) [4, 17]. The proportion of MDR Gram-negative microorganisms was 23% and carbapenem-resistant Gram-negative microorganisms was 18%, similar to the percentage of MDR organisms (12–39%) and the percentage of carbapenem-resistant organisms (16%) in the previous studies [18, 19]. Our study found that the proportion of antibiotic-resistant Gram-positive bacterial infections (MRSA 20%, MRCoNS 70%, and VRE 22.2%) was similar to that in a previous study in the United States (MRSA 30–53% and VRE 14–41%), but higher than the percentage of resistant microorganisms in the study of NARST (MRSA 0.1%, MRCoNS 56%, and VRE 5%) [16, 17]. As a result, the strategies for appropriate antibiotic therapy should target for the increase of antibiotic-resistant bacterial infection.

Viral pathogens diagnosed by laboratory-based confirmation were 15% of the microbiologically documented infection and similar to the previous studies [3, 5]. Viral detection depended on the availability of diagnostic tests. Investigation for viral etiology should be taken into consideration as a source of febrile neutropenia to prevent infectious transmission and reduce the use of empirical antibiotic and antifungal drugs in patients with fever of unknown origin [2].

Fungus is an important causative pathogen in febrile neutropenic patients due to the use of intensive chemotherapy which results in prolonged neutropenia, the use of a central venous catheter, and prolonged use of broad-spectrum antibiotics [20]. Our study found that 12% of febrile neutropenia episodes were caused by fungus. The frequency of fungal infection varied in previous studies between 6 and 18% [3, 5, 6]. The most common isolated pathogens were *Aspergillus* spp. and *Candida* spp., similar to the previous studies [3, 5, 6]. Fungal infection causes significant morbidity and can lead to severe complications because of the difficulty in diagnostic methods and limitations in treatment options [21].

*Mycobacterium tuberculosis* (TB) is a slow-growing microorganism which can cause disease in healthy and immunocompromised patients [22]. Febrile neutropenic patients with impairment of humoral and cellular immunity are susceptible to opportunistic infections including tuberculosis [22]. Our study found a case of extrapulmonary tuberculosis infection. This patient with the first diagnosis of acute myeloid leukemia presented with prolonged fever and prolonged neutropenia for 5 weeks after an induction course of chemotherapy, followed by cough and lymphadenopathy. He was diagnosed with right paratracheal, hilar, and subcarinal lymphadenopathy. Tuberculosis infection in hematological patients may be difficult to diagnose because of atypical presentation and coexistent disease [22]. However, high index of suspicion of TB infection should be a differential diagnosis in febrile neutropenic patients with prolonged fever.

Oropharyngeal infection including oral mucositis is the most common source of infection in clinically documented infection. However, previous studies found that gastrointestinal infection is the most common etiology of febrile neutropenic episodes [2, 4]. The increasing frequency of mucositis is due to an intensive course of chemotherapeutic agents including cytosine arabinoside, prolonged neutropenia, long-term indwelling intravenous catheters, prophylactic antibiotic treatment with fluoroquinolone and cotrimoxazole, and the use of antacids and histamine blockers [21].

In our multivariate analysis, there was no significant risk factor for antibiotic-resistant Gram-negative bacterial infection among the type of underlying malignancy, administered therapy, and remission state of underlying malignancy. The result of our study found that patients with antibiotic-resistant Gram-negative bacterial infection have a significantly longer duration of fever after empirical antibiotic therapy, more severe complications (renal dysfunction), and mortality than non-antibiotic-resistant bacterial infection. The mortality rate within 2 weeks of febrile neutropenia in this study was low at 3.7%, as compared with previously reported mortality rates of 0.6–19.2% in Thailand and other regions of the world [2–5, 23].

The result of our study revealed useful information about the causative pathogens and the incidence of the antibiotic-resistant microorganisms to develop treatment guidelines in febrile neutropenia treatment. However, there were some limitations. First, this study was performed in a single institute. The distribution of causative pathogens and their antibiotic susceptibilities are influenced by the antibiotic resistance status which is varied between each community and hospital. Second, the administered antibiotics and the appropriateness of antibiotic choices for each patient were not determined in this study, which may result in clinical outcomes. The third limitation was the uncertainty of diagnosis in viral infection without laboratory confirmation which might masquerade as fever of unknown origin. Also, since this is a retrospective descriptive study, there were some limitations in data collection.

## 5. Conclusions

Bacteria are the predominant causative pathogen in febrile neutropenic patients. From our study, it was found that Gram-negative bacteria were the most common isolated pathogen. There is an increasing proportion of antibiotic-resistant bacterial infection which is associated with significant morbidity and mortality; therefore, the surveillance of microorganism distribution and strategies for reducing the occurrence of antibiotic-resistant bacterial infection should be established.

## Figures and Tables

**Table 1:** Demographic data of 267 patients with febrile neutropenia.

Patient characteristics	Number (%)
Age, years (median, range)	5.1 (1 month, 15 years)
Male	151 (56.6)
Underlying diseases	
Hematologic malignancy	191 (71.5)
Acute lymphoblastic leukemia	114 (42.7)
Acute myeloid leukemia	44 (16.5)
Lymphoma	20 (7.5)
Other*∗*	13 (4.8)
Solid tumor	76 (28.5)
Brain tumor	23 (8.6)
Neuroblastoma	17 (6.4)
Rhabdomyosarcoma	7 (2.6)
Osteosarcoma	5 (1.9)
Hepatoblastoma	4 (1.5)
Ewing sarcoma	4 (1.5)
Retinoblastoma	4 (1.5)
Others∗∗	12 (4.5)

*∗*Hemophagocytic lymphohistiocytosis, Langerhans cell histiocytosis, chronic myeloid leukemia, juvenile myelomonocytic leukemia; ∗∗Wilms' tumor, primitive neuroectodermal tumor, extrarenal rhabdoid tumor, small-cell lung cancer, soft-tissue sarcoma, atypical teratoid rhabdoid tumor, yolk sac tumor, mix germ cell tumor.

**Table 2:** Characteristics of 563 febrile neutropenia episodes.

Patient characteristics	Total(*n* = 563)
Median (range) duration of fever prior to admission (days)	1 (0, 60)
Interval since the last chemotherapy (days) (median, range)	10 (0, 148)
Therapy preceding infection, *n* (%)	
Induction	155 (27.5%)
Maintenance	68 (12.1%)
Disease status, *n* (%)	
Remission	58 (21.7%)
Not remission	43 (16.1%)
Duration of neutropenia (days) (median, range)	8 (1, 49)
Duration of fever in days (median, range)	3 (1, 38)
White blood cell count on admission (cells/mm³) (median, range)	670 (10, 2, 35, 150)
Absolute neutrophil count on admission (cells/mm³) (median, range)	70 (0, 9, 522)
Antifungal therapy in the past 6 months	123 (21.8%)
G-CSF use before a specific cycle	164 (29.1%)
Steroid in the past 14 days	226 (40.1%)
Central venous catheter	226 (40.1%)
Central line	111 (19.8%)
PICC line	102 (18.1%)
Chemoport	10 (1.8%)
Hickmann	3 (0.5%)

**Table 3:** Etiology and outcome of 563 febrile neutropenia episodes.

Outcome	Total episodes (*n* = 563)
Etiology of fever, *n* (%)	
Fever of unknown origin	279 (49.6%)
Microbiologically documented infection	192 (34.1%)
Clinically documented infection	92 (16.3%)
Oropharyngeal infection	26
Skin and soft-tissue infection	17
Upper respiratory tract infection	16
Lower respiratory tract infection	13
Gastrointestinal tract infection	12
Urinary tract infection	6
CNS infection	2
Clinical complication, *n* (%)	
Oxygen therapy	110 (19.5%)
Shock	100 (17.8%)
ICU admission	78 (13.9%)
Mechanical ventilator use	37 (6.6%)
Renal dysfunction	24 (4.3%)
Hepatic dysfunction	8 (1.4%)
Duration of hospitalization in days (median, range)	25 (1, 163)
Mortality within 2 weeks of a febrile episode	21 (3.7%)

**Table 4:** Causative microorganisms from 214 clinical specimens.

Microorganisms	No.	%
Gram-negative bacteria		
*Escherichia coli*	104	48.6
*Escherichia coli*	38	17.8
*Klebsiella pneumoniae*	19	8.9
*Acinetobacter baumannii*	14	6.5
*Pseudomonas aeruginosa*	12	5.6
*Salmonella*	5	2.3
*Enterobacter* spp.	4	1.9
*Proteus* spp.	3	1.4
*Aeromonas* spp.	2	0.9
Other*∗*	7	3.3
Gram-positive bacteria	50	23.4
Coagulase-negative *Staphylococci*	12	5.6
*Staphylococcus aureus*	10	4.7
*Enterococcus faecalis*	5	2.3
*Bacillus* spp.	5	2.3
Viridans streptococci	4	1.9
*Corynebacterium* spp.	4	1.9
*Streptococcus mitis*	3	1.4
*Enterococcus faecium*	2	0.9
*Enterococcus gallinarum*	2	0.9
Others∗∗	3	1.4
Virus	32	15
Influenza	8	3.7
Parainfluenza	6	2.8
Respiratory syncytial virus	5	2.3
Herpes virus	5	2.3
Varicella virus	4	1.9
Entero/rhinovirus	1	0.5
Human metapneumovirus	1	0.5
Dengue virus	1	0.5
Rotavirus	1	0.5
Fungus		
*Aspergillus* spp.	27	12.6
*Candida* spp.	12	5.6
Yeast cell	12	5.6
*Pneumocystis jirovecii*	2	0.9
*Mycobacterium tuberculosis*	1	0.5

*∗Acinetobacter lwoffii, Burkhoderia cepacia*, *Campylobacter jejuni*, *Klebsiella ozaenae*, *Pseudomonas putida*, *Stenotrophomonas maltophilia*, *Moraxella* spp. ∗∗*Streptococcus bovis*, *Streptococcus constellatus*, and *Micrococcus* spp.

**Table 5:** Summary of febrile neutropenic episodes with polymicrobial infections.

No.	Isolated sites/specimen	Microorganism
1	Blood culture	*Escherichia coli*
	Urine culture	*Proteus* spp.
	Wound	Herpes virus

2	Pus culture	Coagulase-negative *Staphylococcus*, *Enterococcus faecalis*, and *Corynebacterium* spp.
3	Pus culture	*Acinetobacter baumannii* and *Enterococcus* spp.
4	Blood culture	*Escherichia coli* and *Pseudomonas aeruginosa*
5	Blood culture	*Escherichia coli, Bacillus* spp., and *Acinetobacter baumannii*
6	Blood culture	*Klebsiella pneumoniae*
	Tip culture	*Candida parapsilosis*

7	Wound	Herpes simplex virus
	Dengue fever	

8	Blood culture	*Klebsiella pneumoniae* and *Streptococcus bovis*
9	Blood culture	Coagulase-negative *Staphylococcus* and *Staphylococcus aureus*
10	Blood culture	*Bacillus* spp. and *Klebsiella pneumoniae*
11	Blood culture	Coagulase-negative *Staphylococcus* and *Candida parapsilosis*
12	Urine culture	*Candida* spp.
	Tracheal suction culture	*Pseudomonas aeruginosa*

13	Urine culture	*Pseudomonas aeruginosa*
	Nasal swab	Influenza

14	Urine culture	*Pseudomonas aeruginosa*
	Nasal swab	Respiratory syncytial virus

15	Blood culture	*Moraxella* spp.
	Urine culture	*Escherichia coli*

16	Urine culture	*Escherichia coli*
	Nasal swab	Respiratory syncytial virus

17	Nasal swab	Respiratory syncytial virus and entero/rhinovirus
18	Blood culture	*Escherichia coli*
	Urine culture	*Candida tropicalis*

19	Blood culture	*Escherichia coli*
	Urine culture	*Klebsiella pneumoniae*

**Table 6:** Characteristics of febrile neutropenic children with Gram-negative bacterial infection.

Characteristics	Non-antibiotics-resistant bacterial infection (*N* = 84) (%)	Antibiotics-resistant bacterial infection (*N* = 20) (%)	*p* value^+^
Underlying disease			
Hematologic malignancy	65 (77.4)	19 (22.6)	1
Solid tumor	16 (80)	4 (20)	1
Therapy preceding infection			
Induction	19 (22.6)	11 (55)	0.006
Maintenance	7 (8.3)	0 (0)	0.341
Remission stage of underlying			
malignancy			
Complete remission	18 (21.4)	8 (40)	0.093
Noncomplete remission	24 (28.6)	1 (5)	0.038
Central venous catheter	45 (53.6)	13 (65)	0.454
Central line	26 (31)	6 (30)	1
PICC line	16 (19)	5 (25)	0.546
Chemoport	2 (2.4)	0 (0)	1
Hickmann	1 (1.2)	0 (0)	1
Site of infection			
Oropharyngeal infection	1 (1.2)	0 (0)	1
Skin and soft-tissue infection	4 (4.8)	3 (15)	0.127
GI infection	7 (8.3)	0 (0)	0.341
Lower respiratory tract infection	5 (6)	4 (20)	0.066
Upper respiratory tract infection	3 (3.6)	0 (0)	1
Urinary tract infection	25 (29.8)	7 (35)	0.788

*p*+ value calculated by Fisher's exact test.

**Table 7:** Therapeutic response in febrile neutropenic children with Gram-negative bacterial infection.

Characteristics	Non-antibiotics-resistant bacterial infection (*N* = 84)	Antibiotics-resistant bacterial infection (*N* = 20)	*p* value^+^
Fever days after empirical antibiotic therapy (median, range)	7 (1, 36)	15 (8, 23)	0.001
Clinical outcome	24 (28.6%)	10 (50%)	0.109
Oxygen therapy	22 (26.2%)	9 (45%)	0.11
Shock	21 (25%)	8 (40%)	0.266
Admission to the intensive care unit	12 (14.3%)	7 (35%)	0.05
Renal dysfunction	3 (3.6%)	4 (20%)	0.024
Hepatic dysfunction	8 (9.5%)	1 (5%)	0.477
Mechanical ventilator care	2 (2.4%)	5 (25%)	0.124
Duration of hospitalization (median, range)	29 (1, 150)	31 (7, 43)	0.790
Overall 2-week mortality	3 (3.6%)	4 (20%)	0.024

*p*+ value calculated by the Mann–Whitney test or Fisher's exact test as appropriate.

## Data Availability

The data used to support the findings of this study are included within the article.

## References

[1] Santolaya M. E., Alvarez A. M., Avilés C. L. (2007). Admission clinical and laboratory factors associated with death in children with cancer during a febrile neutropenic episode. *Pediatric Infectious Disease Journal*.

[2] Hakim H., Flynn P. M., Knapp K. M., Srivastava D. K., Gaur A. H. (2009). Etiology and clinical course of febrile neutropenia in children with cancer. *Journal of Pediatric Hematology/Oncology*.

[3] Lam J. C., Chai J. Y., Wong Y. L (2015). Causative pathogens of febrile neutropaenia in children treated for acute lymphoblastic leukaemia. *Annals of the Academy of Medicine, Singapore*.

[4] Netbaramee W. (2010). Incidence of febrile neutropenia in pediatric oncologic patients after chemotherapy at King Chulalongkorn memorial hospital.

[5] Traivaree C., Saiwaew A., Lumkul R., Torcharus K. (2012). Causative pathogens in childhood oncologic patients with febrile neutropenia at the Phramongkutklao Hospital. *Royal Thai Army Medical Journal*.

[6] Özdemir Z. C., Koç A., Ayçiçek A. (2016). Microorganisms isolated from cultures and infection focus and antibiotic treatments in febrile neutropenic children from Şanlıurfa, Turkey. *The Turkish Journal of Pediatrics*.

[7] El-Mahallawy H. A., Hassan S. S., El-Wakil M., Moneer M. M., Shalaby L. (2015). Increasing antimicrobial resistance monitored in surveillance analysis of blood stream infections in febrile neutropenic pediatric oncology patients. *Asian Pacific Journal of Cancer Prevention*.

[8] Aslan S., Citak E. C., Yis R., Degirmenci S., Arman D. (2012). Bacterial spectrum and antimicrobial susceptibility pattern of bloodstream infections in children with febrile neutropenia: experience of single center in southeast of Turkey. *Indian Journal of Microbiology*.

[9] González-Barca E., Fernández-Sevilla A., Carratalá J., Grañena A., Gudiol F. (1996). Prospective study of 288 episodes of bacteremia in neutropenic cancer patients in a single institution. *European Journal of Clinical Microbiology & Infectious Diseases*.

[10] Zinner S. H. (1999). Changing epidemiology of infections in patients with neutropenia and cancer: emphasis on gram-positive and resistant bacteria. *Clinical Infectious Diseases*.

[11] Lee J. H., Kim S.-K., Kim S. K. (2016). Increase in antibiotic-resistant gram-negative bacterial infections in febrile neutropenic children. *Infection & Chemotherapy*.

[12] Kim H. S., Park B. K., Kim S. K. (2017). Clinical characteristics and outcomes of *Pseudomonas aeruginosa* bacteremia in febrile neutropenic children and adolescents with the impact of antibiotic resistance: a retrospective study. *BMC Infectious Diseases*.

[13] Freifeld A. G., Bow E. J., Sepkowitz K. A. (2011). Clinical practice guideline for the use of antimicrobial agents in neutropenic patients with cancer: 2010 update by the infectious diseases society of America. *Clinical Infectious Diseases*.

[14] Magiorakos A.-P., Srinivasan A., Carey R. B. (2012). Multidrug-resistant, extensively drug-resistant and pandrug-resistant bacteria: an international expert proposal for interim standard definitions for acquired resistance. *Clinical Microbiology and Infection*.

[15] Turner D., Cheifetz I., Kliegman R. M., Geme J. S. (2020). Shock. *Nelson Textbook of Pediatrics*.

[16] Kosmidis C. I., Chandrasekar P. H. (2012). Management of gram-positive bacterial infections in patients with cancer. *Leukemia & Lymphoma*.

[17] National Antimicrobial Resistance Surveillance, Thailand (NARST) (2019). *Percentage of Susceptible Organisms Isolated from All Specimen, 33 Hospitals*.

[18] Haeusler G. M., Mechinaud F., Daley A. J. (2013). Antibiotic-resistant gram-negative bacteremia in pediatric oncology patients-risk factors and outcomes. *Pediatric Infectious Disease Journal*.

[19] Folgori L., Livadiotti S., Carletti M. (2014). Epidemiology and clinical outcomes of multidrug-resistant, gram-negative bloodstream infections in a European tertiary pediatric hospital during a 12-month period. *Pediatric Infectious Disease Journal*.

[20] Cugno C., Cesaro S. (2012). Epidemiology, risk factors and therapy of candidemia in pediatric hematological patients. *Pediatrics Reports*.

[21] Ozdemir N., Tuysuz G., Celik N. (2016). Febrile neutropenia in children with acute lymphoblastic leukemia: single center experience. *Türk Pediatri Arşivi*.

[22] Chen C. Y., Sheng W. H., Cheng A. (2011). Clinical characteristics and outcomes of *Mycobacterium tuberculosis* disease in adult patients with hematological malignancies. *BMC Infectious Diseases*.

[23] Roongpoovapatr P., Suankratay C. (2010). Causative pathogens of fever in neutropenic patients at King Chulalongkorn memorial hospital. *Journal of the Medical Association of Thailand=Chotmaihet Thangphaet*.

